# Endothelial glycocalyx damage in patients with acute coronary syndrome

**DOI:** 10.1186/cc14660

**Published:** 2015-09-28

**Authors:** Carlos H Miranda, Andre Schmidt, Antônio P Filho, Marcos C Borges

**Affiliations:** 1 Division of Emergency Medicine, Ribeirão Preto School of Medicine, São Paulo University, Ribeirão Preto, São Paulo, Brazil

## Introduction

The endothelial glycocalyx is a thin layer of proteinaceous material at the endothelial surface of the vessels. Damage to this structure can cause protein extravasation and tissue edema, increase platelet and leukocyte adhesion and increase of the shear stress in the vessel due to decreasing the nitric oxide production. On the other hand, a vulnerable atherosclerotic plaque with rupture or erosion characterizes the acute coronary syndrome (ACS). The glycocalyx damage could contribute to this process.

## Objective

To evaluate the glycocalyx damage through syndecan-1 dosage in patients with ACS.

## Methods

We included patients with ACS diagnosis (*n *= 140). These patients had ST-segment elevation myocardial infarction (*n *= 71), were without ST-segment elevation myocardial infarction (*n *= 58) and had unstable angina (*n *= 12). The syndecan-1 level was dosage through commercial ELISA kits (Abcam^®^, Cambridge, UK) at admission. These levels were compared with 45 patients with noncoronary chest pain (NCCP) in which the ACS diagnosis was ruled out through negative electrocardiogram, troponin and imaging test (tomographic or conventional coronary angiography, stress echocardiography) and 24 completely healthy individuals (CONTROL). The values were expressed in median plus percentiles 25 (P_25_) and 75 (P_75_).

## Results

The syndecan-1 levels were higher in the patients with ACS (77 ng/ml; 46-134) compared with the NCCP group (60 ng/ml; 32-79); *p *= 0.01 and CONTROL group (42 ng/ml; 27-80); *p *= 0.001. No difference was observed between the NCCP versus CONTROL group (*p *= 0.83). A syndecan-1 level higher than 148 ng/ml was associated with the ACS diagnosis (relative risk: 1.53; 95 % confidence interval: 1.33-1.80; *p *<0.0001).

## Conclusion

The endothelial glycocalyx damage could contribute to the atherosclerotic plaque vulnerability and the establishment of an ACS.

